# Analysis of the Underlying Mechanism of the Jiu Wei Zhen Xin Formula for Treating Generalized Anxiety Disorder Based on Network Pharmacology of Traditional Chinese Medicine

**DOI:** 10.1155/2022/7761852

**Published:** 2022-05-29

**Authors:** Heng Shao, Quan Gan, Zhuangfei Chen, Shasha Zhu, Yanqing Zhu

**Affiliations:** ^1^Department of Geriatrics, The First People's Hospital of Yunnan Province, Kunming, Yunnan, China; ^2^The Affiliated Hospital of Kunming University of Science and Technology, Kunming, Yunnan, China; ^3^Medical Faculty, Kunming University of Science and Technology, Kunming, Yunnan, China; ^4^Department of Clinical Psychology, The First People's Hospital of Yunnan Province, Kunming, Yunnan, China

## Abstract

Currently, there are many effective pharmacological treatments for generalized anxiety disorder (GAD), formulated herbal granule is also an alternative way. Our research intends to construct a pharmacological network on genetic targets and pathways between Jiu Wei Zhen Xin Formula (JWZXF) and GAD. Through the TCMSP database, we collected the active ingredients of JWZXF and potential targets of the active ingredients. The GAD-related proteins collected from GeneCards database and DisGeNET database were combined. Component-target protein networks were constructed and visualized using Cytoscape 3.8.2 software to comprehensively clarify the relationships between ingredients, components, and targets. The intersection targets were imported into the STRING database, and the protein-protein interaction (PPI) network was constructed. We constructed and analyzed the visualized “drug-target-disease” network. Gene Ontology (GO) enrichment together with Kyoto encyclopedia of genes and genomes (KEGG) enrichment analysis were conducted on the common target through R language. Forty-one effective components and 106 potential targets of JWZXF were found. There were top ten hub genes and multiple important signaling pathways involved in the treatment of GAD with the JWZXF. This study expounded the pharmacological actions and molecular mechanisms of the JWZXF in treating GAD from a holistic perspective. The potential pharmacological effects of the JWZXF are closely related to regulation because not only does it comprehensively analyze the possible mechanism of JWZXF treatment of GAD but it can also facilitate further in-depth research and provide a theoretical basis for the clinical expansion of its application.

## 1. Introduction

Generalized anxiety disorder (GAD) is a chronic mental disorder characterized by excessive tension, worry, and feelings of apprehension that last at least six months. The term GAD was first used in ICD-9; it got the attention of clinical researchers with the publication of DSM-III [1]. Later, DSM-V stated that individuals with GAD might experience restlessness, be easily fatigued, have difficulty concentrating, experience irritability, muscle tension, or sleep disturbance [2]. It is a chronic disease that is prevalent worldwide, with a combined lifetime prevalence of 3.7%, 12 months prevalence of 1.8%, 30 days prevalence of 0.8%, and comorbidity of 81.9%, respectively [3]. In urban China, the prevalence of GAD was 5.3%, with a low diagnosis rate [4]. Currently, many studies that focused on the effective pharmacological treatments of GAD mainly include selective serotonin reuptake inhibitors (SSRIs), serotonin-norepinephrine reuptake inhibitors (SNRIs), quetiapine, agomelatine, benzodiazepines, buspirone, and pregabalin [5, 6]. Among them, escitalopram and paroxetine of SSRIs, and venlafaxine and duloxetine of SNRIs are the most studied, respectively. However, those medicines may not be well tolerated in GAD patients [7], and both SSRIs and SNRIs are associated with decreased efficacy at higher doses [8]. Although there are many recommended pharmacological treatments of GAD, they inevitably have some side effects and may even aggravate treatment-resistant patients. Complementary and alternative medicine (CAM) treatments are now recognized as an efficient alternative treatment [9]. Herbal medicine as a part of CAM is beneficial to the GAD, with an abundance of researchers highlighting the molecular mechanisms, signaling pathways, and neurotrophic factors in mental disorders [10–12]. Novel approaches like pharmacogenetics and pharmacoepigenetics are safer and may improve the treatment response while reducing the socioeconomic burden [13]. A meta-analysis revealed Jiu Wei Zhen Xin Formula (JWZXF) and concluded that a formulated herbal granule is less effective than SSRIs but is safer [14]. This research intends to construct a pharmacological network on JWZXF and GAD genetic targets and reveal their relationship.

## 2. Materials and Methods

### 2.1. Identification and Screening Strategy of Candidate Components in the JWZXF

The components of interest regarding the JWZXF were extracted based on their botanicals, which are *Panax ginseng C. A. Mey* (P.G.), Ziziphi Spinosae Semen (Z.S.S.), *Schisandrae chinensis Fructus* (S.C.F.), *Poria cocos Wolf *(P.C.W.), *Polygala tenuifolia Willd* (P.T.W.), Corydalis Rhizoma (C.R.), *Asparagi radix* (A.R.), *Rehmanniae radix Praeparata* (R.R.P.), and *Cinnanmomi cortex* (C.C.). The total component list of each ingredient was identified from the TCMSP [1] (Traditional Chinese Medicine Systems Pharmacology https://tcmsp-e.com/) database and TCM-ID (TCM-information database https://bidd.group/TCMID/index.html); due to the lack of component information of P.T.W in TCMSP database, we inquired and listed P.T.W component information from TCM-ID. Subsequently, we identified the ADME (absorption, distribution, metabolism, and excretion) properties from the TCMSP database based on the candidate components' information. Currently, drug-likeness (DL) evaluation (e.g., Lipinski's rule of five, Opera's rules of DL, and the ROES filter) is integrated into computational drug design/discovery pipelines. In this study, four main filtering criteria have been implemented in our research and used to screen components that could be involved in the central nervous regulation.

With a DL value higher than 0.18, oral bioavailability value higher than 30%, half-life time longer than four hours, and blood-brain barrier (BBB) penetration rate >0.3 can be retained as candidate components for subsequent analysis [15]. Finally, 41 active components were screened for target predictions within which three common components (CM1, CM2, and CM3), and 106 related targets were identified after removing repetitions, UniProt database (https://www.uniprot.org) was used to convert and calibrate protein names into gene official symbols that are potential genetic targets of the JWZXF.

### 2.2. Disease Target Prediction

With “generalized anxiety disorder” as the keyword, from the databases, GeneCards (https://www.genecards.org/) filtered from its highest “relevance score” to the third quartile that retrieved 1338 items and DisGeNET (https://www.disgenet.org/search, update by May 2020, v7.0) filtered by its “Score_gda” needed to be greater than and equal to 0.8 to help identify 1594 items. The GAD-related proteins collected from the GeneCards and the DisGeNET databases were combined to finally obtain 1977 related genetic targets (without duplication), after using the UniProt database to convert and calibrate gene names to official gene symbols, which are the potential genetic targets of GAD.

### 2.3. Network Construction and Analysis

Combining the result of potential target proteins of the JWZXF from above with the GAD-related proteins, we took the intersection of two datasets and found 50 drugs with shared targets. Component-target protein networks were constructed and visualized using Cytoscape 3.8.2 software to comprehensively clarify the relationships between ingredients, components, and targets. This showed common components of ingredients and off-target components.

The software allows data integration to analyze and visualize complex interactive networks. In these networks, nodes represent components, proteins, pathways, and GAD, while edges represent their interactions.

### 2.4. Construction of the Protein-Protein Interaction (PPI) Network and Analyses of Topological Properties

The intersection targets were imported into STRING (version 11.5 https://string-db.org/cgi/) database, with the “species” set as “Homo sapiens.” Under the condition that the lowest interaction score was equal to 0.400, the TSV result file of protein to protein interactions was obtained, and the PPI network was constructed using the Cytoscape 3.8.2 software. The topological attributes of the PPI network were analyzed, and the value (degree) was calculated, representing the number of connected nodes.

Based on the PPI network, we can use the Cytoscape plug-in to extract subnetwork and hub genes. Cytoscape MCODE plug-in based on the K-core algorithm can be used to find clusters (highly interconnected regions) in a network (degree cutoff = 2, max. Depth = 100, *K*-core = 2 and node score cutoff = 0.2). After extracting the subclusters of PPI, we use the DAVID (version 6.7 October 31, 2020, https://david.ncifcrf.gov/) database to analyze those subcluster interactions and reveal the biological processes of each cluster.

Meanwhile, using CytoHubba plug-in to identify hub genes: we used the maximum cluster centrality (maximum clique centrality, MCC) algorithm to screen hub genes. MCC algorithm integrates 11 topology analysis methods and six centrality analysis methods, which produced high accuracy.

### 2.5. Kyoto Encyclopedia of Genes and Genomes (KEGG) Enrichment Analysis

KEGG is a database that systematically analyzes the metabolic pathways and functions of gene products in cells. The KEGG database helps study genes and express information as a complete network by integrating data from genomes, chemical molecules, and biochemical systems, including metabolic pathways, drugs, diseases, gene sequences, and genomes. We selected the DAVID online database to implement the KEGG enrichment analysis.

The intersection genes were introduced into the DAVID database, the “select identifier” was set to “official gene symbol,” “list type” was set to “gene list,” with the species defined as Homo sapiens, *P*-value ≤0.05 was set as the initial selection threshold. KEGG pathway enrichment analysis was realized, and the ascending order was sorted following the FDR to screen for the top 12 information pathways (ggplot2 package of R).

### 2.6. Gene Ontology (GO) Analysis

To study meaningful functional annotation and biological characteristics of potential targets, GO enrichment analysis was conducted to extract key GO terms (BP: biological process, MF: molecular function, CC: cellular components). In this case, we chose to use the DAVID database to conduct an online analysis that would allow researchers to use the KEGG enrichment together with the GO analysis. The targets, organized and condensed into several functional groups as denoted by their most significant leading term, were visualized in the network. The GO terms that had a *P*-value ≤0.05 were regarded significant and were studied further. Finally, we listed the top 15 GO terms sorted in the ascending order of FDR.

## 3. Results

### 3.1. Screening of the Effective Compounds and Potential Targets of JWZXF

The effective components were extracted based on the criteria we mentioned before, Table 1 lists out the potential effective components from the JWZXF. We also discovered that ingredient C.C did not contain any component that fulfilled our ADME selection criteria.

### 3.2. Component-Target Protein Network Construction and Analysis

**Figure 1:**
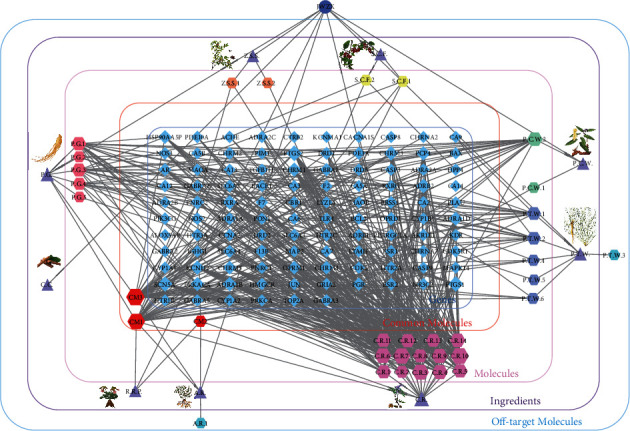
Component-target protein network. The hexagons represent the 41 candidate compounds in the Jiu Wei Zhen Xin formula (JWZXF). The blue diamond represents the genetic names of the target proteins of the nine herbs found by text mining.

We used the data extracted above and Cytoscape 3.8.2 software to build a component-target protein network, which contains 151 nodes (1 formula name, 9 ingredients, 41 effective components, and 106 potential targets of JWZXF), and 528 edges with the size of node based on its degree value is clearly represented in Figure 1. In the central layer of this network, the blue diamond represents target proteins from the center to the outside. Each layer represents common molecules with red hexagons, unique molecules of each ingredient with pink red, orange, yellow, green, lavender, rose red hexagons, ingredients with bluish-purple triangles, and off-target molecules of each ingredient, respectively. From the “common molecules” layer, stigmasterol (CM1) was present in P.G., A.R., and R.R.P.; sitosterol (CM2) was present in C.R., A.R., and R.R.P.; and beta-sitosterol (CM3) was present in P.G. and A.R. From the “molecules” layer, C.R. contains more active components than other ingredients with relatively higher degree values.

### 3.3. Protein-Protein Interaction Network of Targets

**Figure 2:**
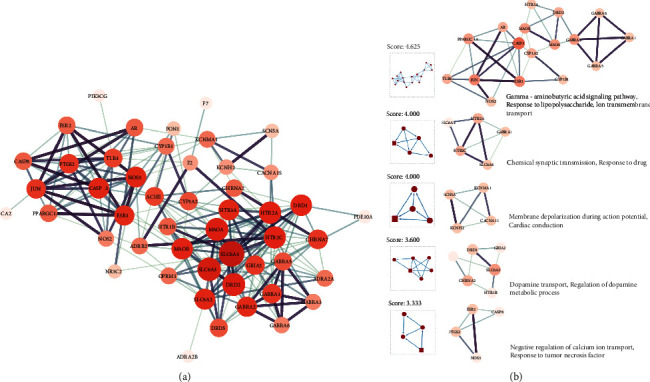
PPI network of JWZXF in the treatment of GAD-related proteins (a) and MCODE analysis of the PPI network and their corresponding GO biological processes (b).

**Figure 3:**
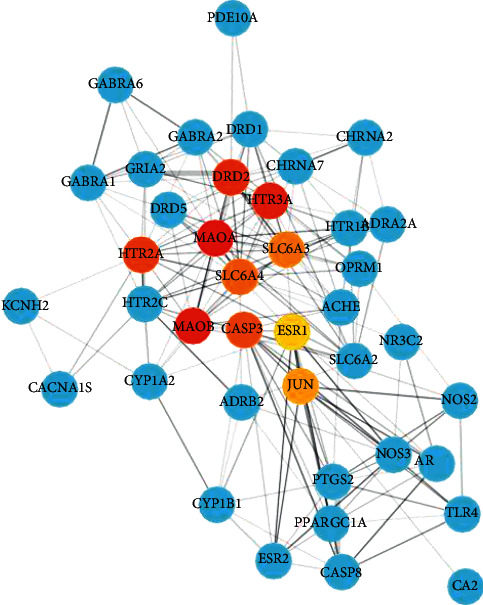
Top 10 hub genes with their neighboring gene interactions. The blue circles are the neighbor gene, red and yellow circles are the hub gene, and the color changes from dark to light according to the MCC score from high to low.

Proteins normally regulate their physiological functions through protein-protein interactions and other pathways. To better reveal the mechanism of the JWZXF in treating GAD, STRING, a database designed to collect and integrate all functional interactions between expressed proteins by integrating known and predicted protein-protein association data from a large number of organisms, was used. The intersection targets obtained above were entered into the STRING database, the PPI data obtained from the STRING database were imported into the software Cytoscape 3.8.2, to construct a PPI network related to GAD (with 49 nodes and 218 edges, one free node was removed) that is represented in Figure 2(a). Then, the Cytoscape MCODE plug-in based on the *K*-core algorithm was used to find clusters (highly interconnected regions) in a network. After calculation, five subcluster interactions were obtained which have network feature scores from 3.333 to 4.625, in the meantime, the DAVID database was used to analyze those subcluster interactions and to reveal the biological process of each cluster represented in Figure 2(b). With the help of Cytoscape CytoHubba plug-in under the algorithm of MCC, the top 10 hub genes found in the PPI network are represented in Table 2. The hub gene interaction with its neighbor genes are represented in Figure 3.

### 3.4. Kyoto Encyclopedia of Genes and Genomes Functional Enrichment Analysis and Gene Ontology Analysis

**Figure 4:**
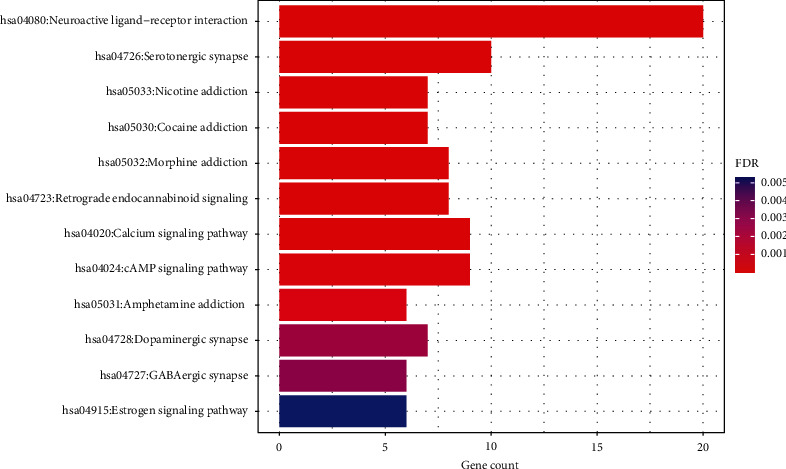
KEGG enrichment analysis of the identified JWZXF in GAD treatment by DAVID database, ranked by an FDR value from low to high.

To macroscopically and comprehensively understand the biological function of the active ingredient target in the JWZXF, we implemented the GO functional enrichment analysis and KEGG pathway enrichment analysis on intersection targets. The results were mapped using the R software as bar plot and bubble plots for both KEGG and GO analysis. The top 12 pathways were screened based on the parameter of counts, as well as in combination with FDR-values in Figure 4, including neuroactive ligand-receptor interaction (hsa04080), serotonergic synapse (hsa04726), nicotine addiction (hsa05033), cocaine addiction (hsa05030), morphine addiction (hsa05032), retrograde endocannabinoid signaling (hsa04723), calcium signaling pathway (hsa04020), cAMP signaling pathway (hsa04024), amphetamine addiction (hsa05031), dopaminergic synapse (hsa04728), GABAergic synapse (hsa04727), and estrogen signaling pathway (hsa04915).

**Figure 5:**
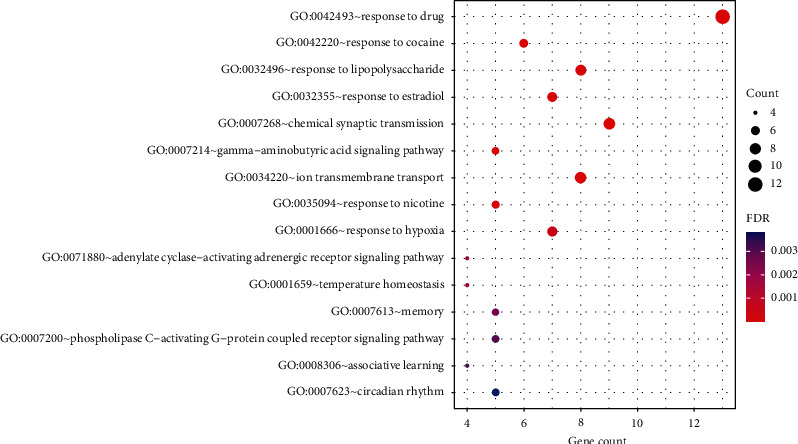
GO enrichment analysis of biological processes showing the top 15 items of 50 identified target proteins by DAVID database according to the FDR value sorted from small to large.

**Figure 6:**
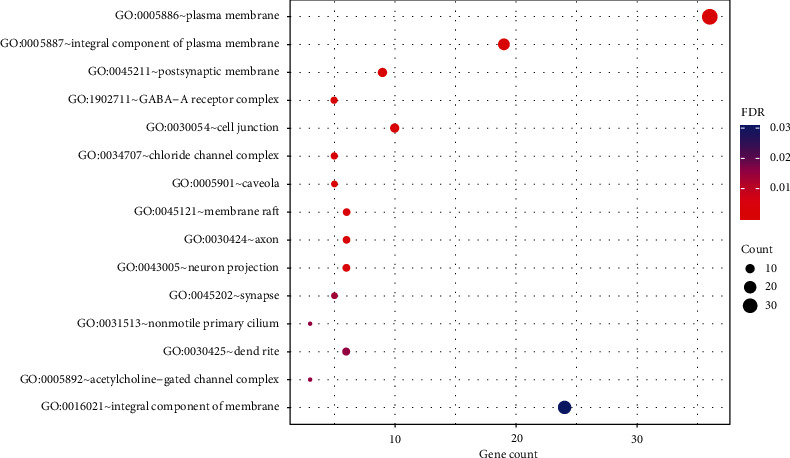
GO enrichment analysis of cellular components showing the top 15 items of 50 identified target proteins by DAVID database according to the FDR value sorted from small to large.

**Figure 7:**
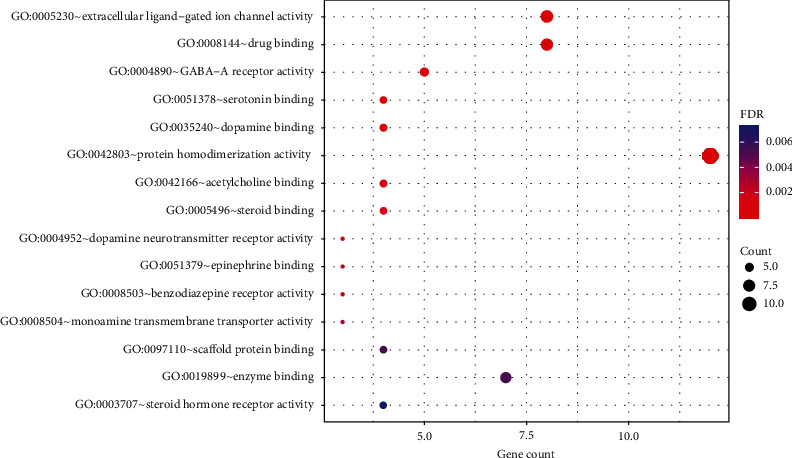
GO enrichment analysis of molecular functions showing the top 15 items of 50 identified target proteins by the DAVID database according to the FDR value sorted from small to large.

The GO analysis showed that the result of BP was significantly enriched in response to drug (GO:0042493), response to cocaine (GO:0042220), response to estradiol (GO:0032355), chemical synaptic transmission (GO:0007268), gamma-aminobutyric acid signaling pathway (GO:0007214), and so on. CC was significantly enriched in the plasma membrane (GO:0005886), integral component of the plasma membrane (GO:0005887), postsynaptic membrane (GO:0045211), GABA-A receptor complex (GO:1902711), cell junction (GO:0030054), and so forth. MF was significantly enriched in extracellular ligand-gated ion channel activity (GO:0005230), drug binding (GO:0008144), GABA-A receptor activity (GO:0004890), dopamine binding (GO:0035240), serotonin binding (GO:0051378), and the like. Based on the FDR value, the top 15^th^ BPs, CCs, and MFs are, respectively, presented in Figures 5–7.

## 4. Summary and Discussion

**Figure 8:**
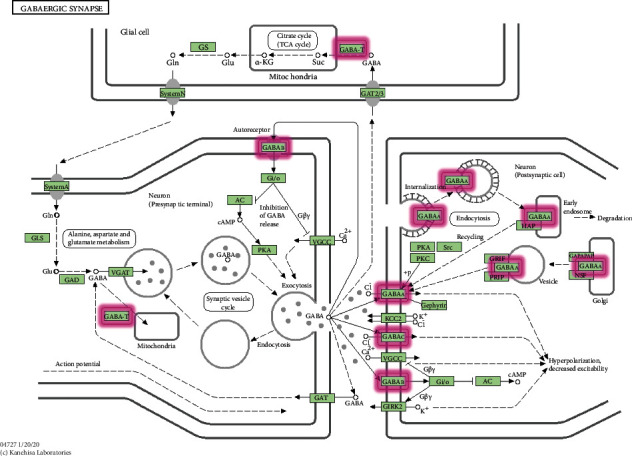
GABAergic synapse. The red rectangle represents the targets related to the component-target-pathway network, by Kanehisa Laboratories (https://www.kegg.jp/pathway/map04727).

Establishing the PPI network of the JWZXF and GAD revealed different pathways, as Figure 2(b) demonstrates that GABA signaling is a crucial pathway. The prolonged *γ*-aminobutyric acid (GABA) transmitter regulatory dysfunction in the animal and human brain contributes to anxiety disorders, especially in limbic systems. Brain circuits in the amygdala are thought to contain an inhibitory network of gamma-aminobutyric acid (GABAergic) interneurons, and thus this neurotransmitter plays a key role in regulating anxiety responses in both normal and pathological states [16]. Reducing GABA-mediated inhibition is one of the effective methods available for the modulation of neuronal excitability [17]. Many researchers have expressed great interest in developing novel medications acting on different subtypes of GABA receptors to manage GAD [18, 19] (Figure 8). Significantly, in second place are the pathways of chemical synaptic transmission and membrane depolarization during the action potential. The excitability and modulation of nerve synapses and the effective release of neurotransmitters also act on anti-anxiety [20, 21]. Psychiatric disorders are closely related to synaptic genes; additionally, mutations of the synaptic genes are also considered as risk factors for mental illness [22]. Moreover, GAD is mostly associated with peripheral inflammatory responses and changes in synaptic transmission profoundly affect these inflammatory responses [23]. Depolarization of the cell membrane potential can cause the alteration of neural connectivity, circuit, and neuron growth, which leads to neurological diseases [24]. Changes in the membrane potential that regulate brain metabolism can cause a series of pathophysiological processes, homeostatic alteration effectively acts on the whole body to reduce anxiety disorder [24, 25]. Moreover, evidence reveals that neuropsychiatric diseases bring about a dysregulation of the dopamine system and pathways [26].

Through the PPI network, the influence and effect of the JWZXF on several important pathways of GAD was observed. Many classical antipsychotic medications have a regulatory effect on the GABA pathway, and the JWZXF primarily acts on this pathway as well. Many studies now focus on finding new medications that selectively act on GABA subunit receptors of benzodiazepine (BZD) anxiolytics that have obvious side effects [18]. We must consider whether the active ingredients of JWZXF action GABA with a broad or selective spectrum are worthy of further discussion. As previously mentioned, selected active ingredients can all pass through the BBB which act on the central nervous system, but the peripheral regulation cannot be ignored in the treatment of GAD. In the future, more experiments and research should be conducted by applying JWZXF to influence both central nervous system and peripheral nerves.

Figure 3 depicts the relationship among hub genes and their neighbors, as Table 2 reveals that the top 10 hub genes ranked by MCC sores in the PPI network are *MAOA*, *MAOB*, *HTR3A*, *DRD2*, *HTR2A*, *CASP3*, *SLC6A4*, *SLC6A3*, *JUN*, and *ESR1*, respectively. GAD is a heritable disorder with a risk of series genes [27]. In the psychotherapy-epigenetic aspect, JWZXF is more involved in the monoamine oxidase genes, which are key enzymes to degrade neurotransmitters, and levels of *MAOA* gene methylation may be related to the categories and severity of the neuropsychiatric disorder [28, 29]. The activity genotype of the *MAOA* gene also correlates with emotional stability, impulse control, and emotion control [30]. *HTR3A* and HTR3B genes, which code subunits of serotonin receptors and their polymorphisms, may serve as predictors of 5-HT_3_ antagonists and SSRIs as well [31–33]. Some researchers demonstrated that the loss of the *HTR3A* gene or *HTR3A* inactivation can induce anxiolytic-like features that have comparability of 5-HT_3_ antagonists [34]. More interestingly, anxiety disorder and pain share the same pathway that gives us more chance to focus on the polymorphism of the *HTR3A* gene to manage chronic pain [31, 35, 36]. *DRD2* is also a risk gene related to GAD and may predict mental disorders at an early age [37, 38]. *SLC6A4* gene, which is a serotonin transporter gene, encodes a membrane protein through the transportation of serotonin to play a role in GAD [39, 40]. Additionally, caspase-3 action on neuronal metabolism is also significant to GAD [41].

The hub gene targets associated with the active components of the JWZXF mainly act on monoamine oxidase and serotonin receptors, especially selective 5-HT_3_ receptors. It is difficult to determine how the JWZXF affects the pharmacokinetics of monoamine oxidase; so, JWZXF may have a monoamine oxidase inhibitor-like component. Drug interactions between the JWZXF and other drugs should be looked into [42, 43]. However, for patients with anxiety and chronic pain, such as fibromyalgia, JWZXF can regulate pain by acting on the HTR3 gene, making it a good choice when GAD is combined with chronic pain.

**Figure 9:**
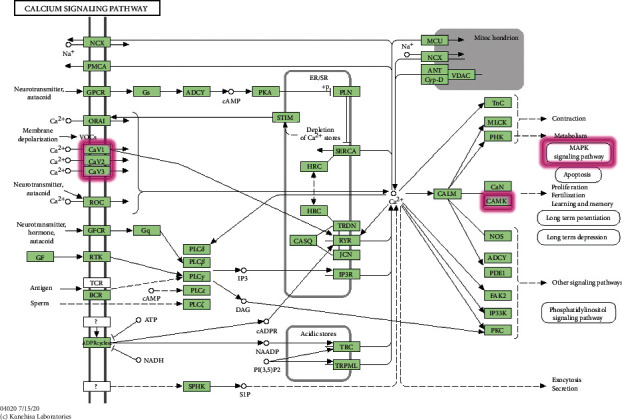
Calcium signaling pathway. The red rectangle represents the targets related to the component-target-pathway network, by Kanehisa Laboratories (https://www.kegg.jp/entry/map04020).

The KEGG pathway enrichment analysis showed multiple signaling pathways involved in the treatment of GAD with JWZXF. Particularly, the neuroactive ligand-receptor interaction, serotonergic synapse, calcium signaling pathway, and the cAMP signaling pathway, in which JWZXF-associated hub gene targets closely related to GAD were enriched. The neuroactive ligand-receptor interaction triggers intracellular signaling that modulates important gene expression dedicated to neural plasticity and stress processing that leads to emotional disorders [44, 45]. A higher level of the neuroactive ligand-receptor interaction gene expression can reduce anxiety-related behavior [46]. Through up- and downregulated signal pathways in the prefrontal cortex, neuroactive ligand-receptor interaction and serotonergic synapse may relate to the susceptibility and resilience of stress [47]. Notably, neurotransmitters also act on different calcium pathways which channels Cav 1.2 and Cav 1.3 are key signal pathways in neuro. Calcium activates calcium senor calmodulin, then, Ca^+^ combines with CaM-dependent protein kinases. The Ca^+^/CaM-dependent protein kinases is activated in a successive way. Additionally, cascade pathways of Ras/mitogen-activated protein kinase and cAMP-responsive element-binding protein (CREB) to make gene expression are related to neuronal plasticity and GAD [48, 49] (Figure 9).

## 5. Conclusion

This study investigated the molecular mechanism of the JWZXF in the treatment of GAD by establishing multiple network models. Studies have shown that the most effective ingredient of the JWZXF in treating GAD is Corydalis, and the most involved compounds may be stigmasterol, sitosterol, and beta-sitosterol. The potential pharmacological effects of the abovementioned active compounds are closely related to regulated GABA receptors, dopamine receptors, chemical synaptic transmission, and membrane depolarization during action potential. Gene *MAOA*, *MAOB*, *HTR3A*, *DRD2*, *HTR2A*, *CASP3*, *SLC6A4*, and *SLC6A3* are possible targets of treatment by the JWZXF. Additionally, JWZXF may primarily modulate the neuroactive ligand-receptor interaction, serotonergic synapse, calcium signaling pathway, and cAMP signaling pathway. The network pharmacology analysis of JWZXF treatment of GAD is of great significance. Not only does it holistically analyze the possible mechanism of JWZXF treatment of GAD, it also facilitates further in-depth research and provide a theoretical basis for the clinical expansion of its application.

## Figures and Tables

**Table 1:** Active components identified from nine herbs.

Ingredients	Number	Components (abbreviations)
*Panax ginseng C. A. Mey.* (P.G.)	7	Stigmasterol (CM1), Beta-sitosterol (CM3), Inermin (P.G.1), Arachidonate (P.G.2), Frutinone A (P.G.3), Girinimbin (P.G.4), Alexandrin_qt (P.G.5)
Ziziphi Spinosae semen (Z.S.S.)	2	Daucosterol (Z.S.S.1), Phytosterol (Z.S.S.2)
*Schisandrae chinensis fructus* (S.C.F.)	2	Angeloylgomisin O (S.C.F.1), Wuweizisu C (S.C.F.2)
*Poria cocos (schw.) wolf.* (P.C.W.)	2	Ergosta-7,22e-dien-3beta-ol (P.C.W.1), Hederagenin (P.C.W.2)
*Polygala tenuifolia Willd* (P.T.W.)	6	4-Methoxycinnamic acid (P.T.W.1), Onjixanthone I (P.T.W.2), Perlolyrine (P.T.W.3), Trans-asarone (P.T.W.4), 1,7-Dihydroxyxanthone (P.T.W.5), Trans-asarone (P.T.W.6)
*Corydalis rhizoma* (C.R.)	16	Stigmasterol (CM1), Sitosterol (CM2), Berberine (C.R.1), Coptisine (C.R.2), Cryptopin (C.R.3), Dihydrochelerythrine (C.R.4), Dihydrosanguinarine (C.R.5), Cavidine (C.R.6), (R)-canadine (C.R.7), (-)-alpha-N-methylcanadine (C.R.8), Dehydrocavidine (C.R.9), Leonticine (C.R.10), 24240-05-9 (C.R.11), Stylopine (C.R.12), Tetrahydrocorysamine (C.R.13), C09367 (C.R.14)
*Asparagi radix* (A.R.)	4	7-Methoxy-2-methylisoflavone (A.R.1), Stigmasterol (CM1), Sitosterol (CM2), Beta-sitosterol (CM3),
*Rehmanniae radix praeparata* (R.R.P.)	2	Stigmasterol (CM1), Sitosterol (CM2)
*Cinnanmomi cortex* (C.C.)	0	—

**Table 2:** Top 10 hub genes ranked by MCC scores in the PPI network.

Ranks	Gene symbol	Score
1	*MAOA*	1536
2	*MAOB*	1394
3	*HTR3A*	1260
4	*DRD2*	1220
5	*HTR2A*	1038
6	*CASP3*	796
7	*SLC6A4*	717
8	*SLC6A3*	691
9	*JUN*	673
10	*ESR1*	629

## Data Availability

The dataset generated for this study is available on request to the corresponding author.

## Abbreviations

GAD: Generalized anxiety disorder
SSRIs: Selective serotonin reuptake inhibitors
SNRIs: Erotonin-norepinephrine reuptake inhibitors
CAM: Complementary and alternative medicine
JWZXF: Jiu Wei Zhen Xin formula
P.G.: Panax ginseng C. A. Mey
Z.S.S.: Ziziphi spinosae semen
S.C.F.: Schisandrae chinensis fructus
P.C.W.: Poria cocos wolf
P.T.W.: Polygala tenuifolia willd
C.R.: Corydalis rhizoma
A.R.: Asparagi radix
R.R.P.: Rehmanniae radix praeparata
C.C.: Cinnanmomi cortex
TCMSP: Traditional Chinese Medicine Systems Pharmacology
TCM–ID: TCM-Information database
ADME: Absorption, distribution, metabolism, and excretion
DL: Drug-likeness
BBB: Blood-brain barrier
PPI: Protein-protein interaction
MCC: Maximum clique centrality
KEGG: Kyoto encyclopedia of genes and genomes
GO: Gene ontology
BP: Biological process
MF: Molecular function
CC: Cellular components
CM1: Stigmasterol
CM2: Sitosterol
CM3: Beta-sitosterol
P.G.1: Inermin
P.G.2: Arachidonate
P.G.3: Frutinone
P.G.4: Girinimbin
P.G.5: Alexandrin_qt
C.R.1: Berberine
C.R.2: Coptisine
C.R.3: Cryptopin
C.R.4: Dihydrochelerythrine
C.R.5: Dihydrosanguinarine
C.R.6: Cavidine
C.R.7: (R)-Canadine
C.R.8: (-)-Alpha-N-methylcanadine
C.R.9: Dehydrocavidine
C.R.10: Leonticine
C.R.11: 24240-05-9
C.R.12: Stylopine
C.R.13: Tetrahydrocorysamine
C.R.14: C09367 (C.R.14)
P.T.W.1: 4-Methoxycinnamic acid
P.T.W.2: Onjixanthone I
P.T.W.3: Perlolyrine
P.T.W.4: Trans-asarone
P.T.W.5: 1,7-Dihydroxyxanthone
P.T.W.6: Trans-asarone
A.R.1: 7-Methoxy-2-methylisoflavone
P.T.W.1: 4-Methoxycinnamic acid
P.T.W.2: Onjixanthone I
P.T.W.3: Perlolyrine
P.T.W.4: Trans-asarone
P.T.W.5: 1,7-Dihydroxyxanthone
P.T.W.6: Trans-asarone
P.C.W.1: Ergosta-7,22E-dien-3beta-ol
P.C.W.2: Hederagenin
Z.S.S.1: Daucosterol
Z.S.S.2: Phytosterol
S.C.F.1: Angeloylgomisin O
S.C.F.2: Wuweizisu C
GABA: *γ*-Aminobutyric acid
BZD: Benzodiazepine
CREB: CAMP-responsive element-binding protein.
